# Lésions verruqueuses périnéales

**DOI:** 10.11604/pamj.2017.28.98.13844

**Published:** 2017-09-29

**Authors:** Ilhame Naciri, Baderddine Hassam

**Affiliations:** 1 Service de Dermatologie et Vénérologie, Centre Hospitalier Universitaire Ibn Sina, Faculté de Médecine et de Pharmacie, Université Mohammed V, Rabat, Maroc

**Keywords:** Lichen plan, verruqueux, hypertrophique, périnéal, Lichen planus, verrucous, hypertrophic, perineal

## Abstract

Verrucous lichen planus (VLP), is a chronic inflammatory dermatosis mainly involving the lower limbs. It rarely affects the perineal area, where it occurs in association with other mucocutaneous lichen lesions. We here report the case of a 51-year old patient with isolated perineal verrucous lichen planus. The patient, who had no significant medical history, presented with painless, verrucous, slightly itchy lesions in the perineal area that had gradually increased in volume over the past 8 years. Clinical examination revealed the presence of multiple firm grayish budding lesions with a rough surface, measuring 1-4 cm in diameter, in the perianal and scrotal area (A). The remainder of the somatic examination was normal. Histology showed verrucous acanthotic epidermis associated with mainly lymphocytic inflammatory infiltrate penetrating the basement membrane and pigmentary incontinence with the formation of apoptotic bodies in the basal layer of epidermis, without signs of viral infection or malignant transformation (B). The diagnosis of verrucous lichen planus was retained. Hepatitis C serology, lipid assessment as well as abdominal ultrasound were normal. A very strong local corticosteroid therapy was prescribed. The patient had a favorable outcome without recidivism at 18-months’ follow-up.

## Image en médecine

Le lichen plan verruqueux (LPV), est une dermatose inflammatoire chronique, qui siège électivement au niveau desmembres inférieurs. La localisation périnéale est rare, souvent associée à d’autres lésions de lichen cutanéo-muqueux. Nous rapportant un cas à localisation périnéale isolée. Il s’agit d’un patient âgé de 51 ans, sans antécédent pathologique notable, qui consultait pour des lésions verruqueuses indolores, légèrement prurigineuses, au niveau périnéal, augmentant progressivement de taille depuis 8 ans. L’examen clinique révélait la présence de multiples lésions bourgeonnantes, fermes, grisâtres à surface rugueuse, de 1 à 4 cm de diamètre, siégeant au niveau péri anal et scrotal (A). Le reste de l’examen somatique était sans particularité. L’étude histologique montrait un épiderme acanthosique d’aspect verruqueux, associé à un infiltrat inflammatoire à prédominance lymphocytaire grignotant la membrane basale, et une incontinence pigmentaire avec des corps apoptotiques au niveau de l’assise basale épidermique, sans objectiver de signes d’infection virale ou de transformation maligne (B). Le diagnostic d’un lichen plan verruqueux était retenu. La sérologie de l’hépatite C, le bilan lipidique, ainsi que l’échographie abdominale étaient sans anomalies. Une corticothérapie locale classe très forte a été prescrite. L’évolution était favorable sans récidive, avec un recul de 18 mois.

**Figure 1:**
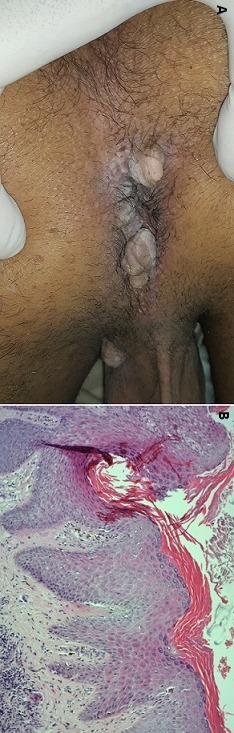
A) multiples lésions bourgeonnantes à surface rugueuse, siégeant au niveau péri anal et scrotal; B) histologie cutanée (ColorationHE, G x 4), épiderme acanthosique, surmonté par une hyperkératose orthokératosique, associé à un infiltrat inflammatoire à prédominance lymphocytaire, grignotant la membrane basale

